# Brewery spent grain derived carbon dots for metal sensing

**DOI:** 10.1039/d2ra00048b

**Published:** 2022-04-14

**Authors:** Aurel Thibaut Nkeumaleu, Daniele Benetti, Imane Haddadou, Michael Di Mare, Claudiane M. Ouellet-Plamondon, Federico Rosei

**Affiliations:** École de technologie supérieure, Université du Québec 1100 Notre-Dame West Montréal H3C 1K3 Canada Claudiane.Ouellet-Plamondon@etsmtl.ca; INRS 1650 Boulevard Lionel-Boulet Varennes QC J3X 1P7 Canada daniele.benetti@inrs.ca federico.rosei@inrs.ca

## Abstract

This article presents a proof-of-concept to recycle microbrewery waste as a carbon source for synthesizing carbon dots (CDs). A simple method has been developed to synthesize water-soluble CDs based on microwave irradiation of brewery spent grain. The structures and optical properties of the CDs were characterized by ultraviolet-visible (UV-Vis) spectroscopy, photoluminescence spectroscopy (PL), X-ray photoelectron spectroscopy (XPS) and transmission electron microscopy. The effects of reaction time, temperature and pH on the properties of carbon dots were studied. These CDs were found to be spherical with an average diameter of 5.3 nm, N-doped, containing many functional groups (hydroxyl, ethers, esters, carboxyl and amino groups), and to exhibit good photoluminescence with a fluorescent quantum yield of 14%. Finally, the interaction between carbon dots and metal ions was investigated towards developing CDs as a sensing technology for water treatment, food quality and safety detection.

## Abstract 2

This article presents a proof-of-concept to recycle microbrewery waste as a carbon source for synthesizing carbon dots (CDs).

## Graphical abstract

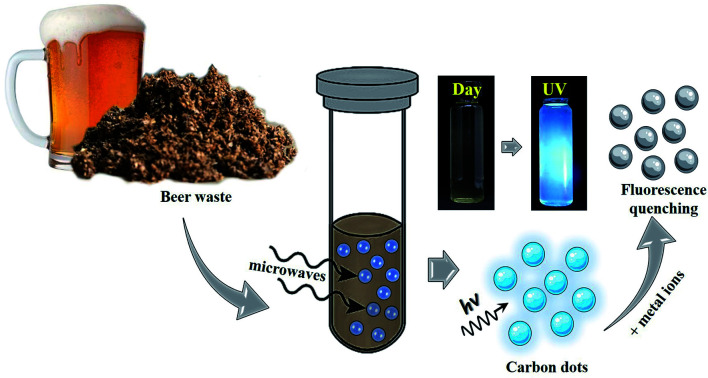

## 1 Introduction

Carbon dots (CDs) are carbon nanoparticles with a size usually smaller than 10 nm. Their structure is characterized by multiple functional groups, such as hydroxyl and carboxyl, and a conjugated core with mixed sp^3^ and sp^2^ bonds.^1–3^ In terms of composition, they are mostly made of carbon, with some oxygen, hydrogen and nitrogen, the four most abundant elements on planet Earth. Their high photoluminescence quantum yield (PL QY), photostability, and low toxicity make them valuable alternatives to inorganic, heavy metal-based quantum dots (QDs).^1,4^ CDs have been used successfully in a variety of optoelectronic applications such as bio- and chemo-sensors, water splitting, LEDs, solar cells and luminescent solar concentrators.^5–11^ Among all those applications, their use as sensing nanomaterials is the most widely reported. CDs can effectively detect a large variety of analytes (ions, small and macromolecules, cells, micro-organisms).^12^ Heavy metals and other contaminants affect the water quality, the environment and public health. In this regard, CDs are successfully employed to detect and quantify some metal ions such as Cu^2+^, Co^2+^, Fe^3+^, Pb^2+^.^13,14^ These fluorescent probes provide a rapid, sensitive and straightforward detection while the standard methods are long, expensive, and need sophisticated instruments.^15^

CDs can be produced from top–down or bottom–up approaches using various synthetic methods, such as hydrothermal, laser ablation, and arc discharge.^16^ In top–down processes, CDs are formed by breaking larger hydrocarbons into the desired nanoparticles. In bottom–up approaches, they are obtained by assembling molecular precursors. Several studies have highlighted the possibility of synthesizing CDs from biosources, such as milk, lemon juice, and orange peels.^2,17,18^ CDs can also be synthesized from by-product biosources and waste products.^19,20^

Brewer's waste grain is generated in large quantities during beer fermentation, consisting primarily of cereal by-products from the distillation process.^21^ These by-products are lignocellulosic biomass materials formed by cellulose, non-cellulosic polysaccharides, and lignin.^22^ Its biodegradability creates several opportunities for value-added products such as dietary supplements in animal feed, sources for various yeast extracts, and bioconversion feedstock.^23^ The adoption of more robust environmental protection laws has increased the cost of beer production, prompting renewed interest in recycling beer waste.^24,25^ The present investigation proposes an alternative method to reduce waste and add value to beer production by exploiting this carbon-rich source as a raw material for synthesizing CDs. To our knowledge, there is no previous report on the use of this lignocellulosic biomass waste to produce CDs.

The optical properties of CDs are also affected by the use of heteroatomic dopants, such as nitrogen, sulphur, boron and phosphorus.^26–29^ These additives can promote a favorable electronic structure, which improves the fluorescence intensity. The addition of heteroatomic dopants, such as nitrogen, provides an opportunity to efficiently tune the intrinsic fluorescence properties of CDs.

Several recent studies focused on the synthesis and application of CDs based on a variety of carbon sources, including biomass, sugar and sugarcane molasses.^30–32^ Most of these works used the hydrothermal method, which is inexpensive but requires a long reaction time. This study shows a proof-of-concept of a microwave method for quickly and easily synthesizing CDs while simultaneously valorizing a large volume industrial waste in a perspective of a circular economy. The influence of the pH, temperature and reaction time on the optical properties of brewery waste-derived CDs were investigated. Furthermore, the obtained CDs are used as optical sensors for metal ions detection, which are a significant source of water pollution, by the principle of fluorescence quenching.^33^ By this technique, the metal ion concentration in the solution can be efficiently correlated to fluorescence intensity through spectroscopic measurements. Further studies will be conducted to provide additional characterization of these brewery-based CDs as well as to develop new practical methods for utilizing them.

## 2 Experimental

### 2.1 Chemicals and materials

Brewery waste was provided by “Brasseurs de Montréal”, Montreal, Canada. Total organic carbon, total phosphorus, and total ammoniacal nitrogen were determined according to the standard methods, and the results are summarized in Table 1.^34^ Pure chemical compounds were purchased from Sigma Aldrich. The article is written from an environmental engineering perspective and the detailed chemical composition is usually not analyzed.

**Table 1:** Physicochemical parameters of 10 g l^−1^ of grinded brewery waste

Parameters component	Concentrations (mg l^−1^)
Total organic carbon	330.2
Total nitrogen	121.2
Ammonia nitrogen	9.4
Total phosphorus	82.3
Chemical oxygen demand	3522
Suspended solids (mg g^−1^)^35^	127.4

### 2.2 Synthesis of carbon dots

As received from the brewery plant, the brewery waste was carbonized in the microwave in the presence of air for a total of 15 minutes in 3 sets of 5 minutes at 200 °C. A stable black powder was obtained after grinding the resulting deposit in a mortar. This carbonization pretreatment is essential to improve the yield of CDs production and safely store the brewery waste at room temperature. Each CD sample was prepared by mixing 5 g of the obtained black powder with 10 ml of distilled water. The solution was then put in a microwave reaction vial (volume 50 ml) and subjected to microwave radiation. After preliminary tests, two series of comparative samples were treated with alternative microwave temperatures (200 °C, 250 °C, and 300 °C) and time combinations (10, 15, 20, and 30 min). The first set of samples was irradiated for 10 min at 200 °C, 250 °C, and 300 °C, while for the second, the temperature was fixed at 250 °C and irradiated for different durations of 10, 15, 20, and 30 minutes. After microwave irradiation, a black solution was obtained. The solution was centrifuged at 12 000 rpm for 20 min. The supernatant was kept and passed through a 0.2 μm cellulose filter membrane and dialyzed for 24 hours with a 1 kDa dialysis bag. The solution inside the bag was then freeze-dried for 24 hours to obtain a CD-rich powder that can be stored for future characterization measurements.

### 2.3 Instruments and characterization of carbon dots

The microwave oven used was a CEM microwave reactor model 909480. The centrifuge was a Thermofisher Sorvall ST 16. The freeze dryer used was a Labconco model 73820 series. High-resolution transmission electron microscopy (HRTEM) images were acquired using an FEI Titan Krios 300 kV Cryo-STEM. Sample particle sizes were characterized by Transmission Electron Microscopy (TEM) using a JEOL 2100F. A drop of the suspension was deposited on the carbon grid to prepare the samples for detection under TEM. Absorption spectra for each CD sample (1 mg ml^−1^) were acquired with a CARY300 Bio UV spectrophotometer. A Cary Eclipse was used to evaluate the fluorescence spectra of each sample (*λ*_ex_ = 350 nm and 400 nm). The solvent solution used was primarily distilled water. To characterize the functional groups present on the surface of CDs, Fourier-transformed infrared (FT-IR) spectra were collected with VARIAN 3100 Excalibur-series FTIR spectrometer. X-ray photoelectron spectroscopy (XPS) was performed in a VG Escalab 220i-XL equipped with hemispherical analyzer, applying a Twin Anode X-Ray Source, calibration by carbon at 284.6 eV.

### 2.4 Quantum yield measurement

The quantum yield (QY) of the brewery waste-derived CD synthesized at 250 °C for 10 min in distilled water (pH 7) was calculated by measuring the integrated photoluminescence intensity in water (refractive index: 1.33) against quinine sulphate in H_2_SO_4_ (refractive index 1.33, quantum yield 0.54 at 360 nm).^36^ The formula used is:

1 *φ*_CD_ = *φ*_QS_ × (*I*_CD_/*I*_QS_) × (*η*_CD_^2^/*η*_QS_^2^)

where *φ*, *I* and *η* represent the quantum yield, the slope of integrated PL intensity and the refractive index, respectively.

### 2.5 Metal sensor testing procedure

Empirical calibration relationships were generated for Cu^2+^ and Fe^3+^ ions in solution with the CDs by fluorescence spectroscopy. The procedure consisted of gradually varying the Fe^3+^ or Cu^2+^ ion concentration in 2 ml of a solution produced with 1 mg ml^−1^ of CDs. The fluorescence intensity was measured for 0, 100, 200, 300, 400 and 500 μM concentrations, and a calibration curve was built for both Cu and Fe ions.

## 3 Results and discussion

### 3.1 Synthesis and characterization of brewery waste-derived CDs

**Fig. 1:**
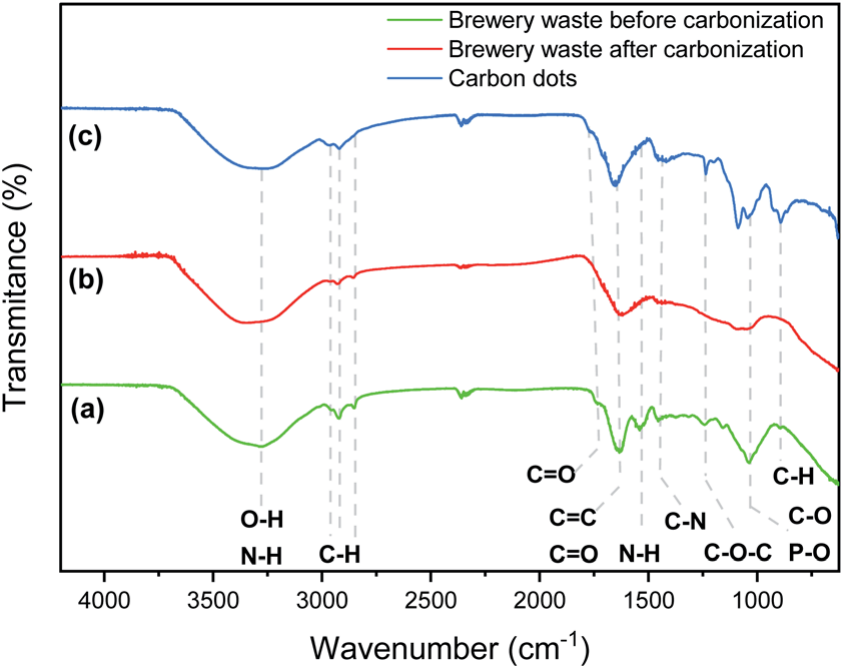
FTIR spectra of the brewery waste before (green) (a) and after the thermal treatment (red) (b), the CDs (blue) (c).

The microbrewery waste was mainly composed of an organic component with a significant content of phosphorous and nitrogen (Table 1). This is a desirable quality for the targeted application as nitrogen and phosphorus are suitable dopants to promote the fluorescence of CDs. Nitrogen is the most frequent doping element used to increase the quantum yield, while phosphorous can modulate and tune the optical features of the CD.^26,29^ The brewery wastes are organic matter composed of protein, carbohydrates, fibres, some phenolic compounds and a small number of lipids.^37^ As visible in the FTIR spectrum, the brewery waste presents the characteristic peaks of carbohydrates and proteins (Fig. 1a).^38^ The broad band around 3200–3300 cm^−1^ corresponds to the stretching vibrations of O–H and N–H groups.^39,40^ The intense band at 1760–1690 cm^−1^ is assigned to aromatic skeletal vibrations (C=C) and the carbonyl stretch (C=O) of ketone and carboxylic acid group.^26^ The peak at 2927, 2853 and 1040 cm^−1^ correspond to the stretching vibrations of C–H and H–C=O as well as C–O and P–O, respectively. In addition, the peak at 1530 and 1457 cm^−1^ are attributed to –NH deformation of amide II and aromatic C–N heterocycles, respectively. The small band at 1243 cm^−1^ corresponds to C–O–C stretching vibrations in polysaccharides.^41^ After thermal treatment of the brewery waste (Fig. 1b), the bands corresponding to –NH deformation of amide II and to C–O–C stretching vibrations in polysaccharides disappeared. On the other hand, FTIR spectra of the synthesized CDs showed similar absorption peaks to their precursor with more substantial peaks observed at 1652, 1417, 1238, 1085, 1046 and 890 cm^−1^ assigned to C=O/C=C, C–N, C–O–C, C–O and C–H/P–O–H bending, respectively (Fig. 1c).^42^ The prominent peak at 3260 cm^−1^ clearly indicated the presence of O–H and N–H groups. Overall, the FTIR spectra validate the graphitic structure of CDs owing to the presence of C=C peak, whereas C=O, C–O, and OH peaks indicate the presence of hydroxyls, carboxylic and amino groups in the surface of the CDs.

**Fig. 2:**
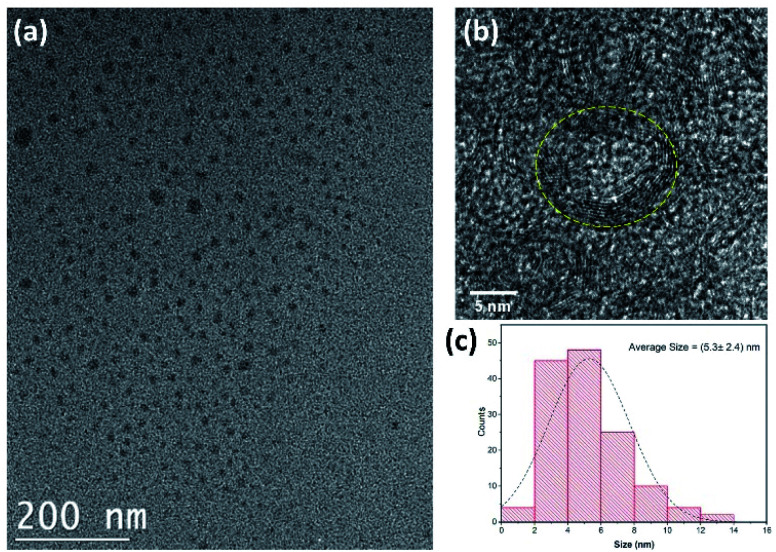
(a) TEM and (b) HRTEM image of carbon quantum dots. (c) The size distribution of CDs. (CDs were prepared at 250 °C for 10 min).

The morphological characterization of the CDs was performed by TEM, revealing the irregular circular shapes of CDs with a uniform distribution (Fig. 2a and b). The average size of the as-prepared CDs was found to be 5.3 ± 2.4 nm (Fig. 2c). This value is consistent with most previous findings in the literature.^43,44^

**Fig. 3:**
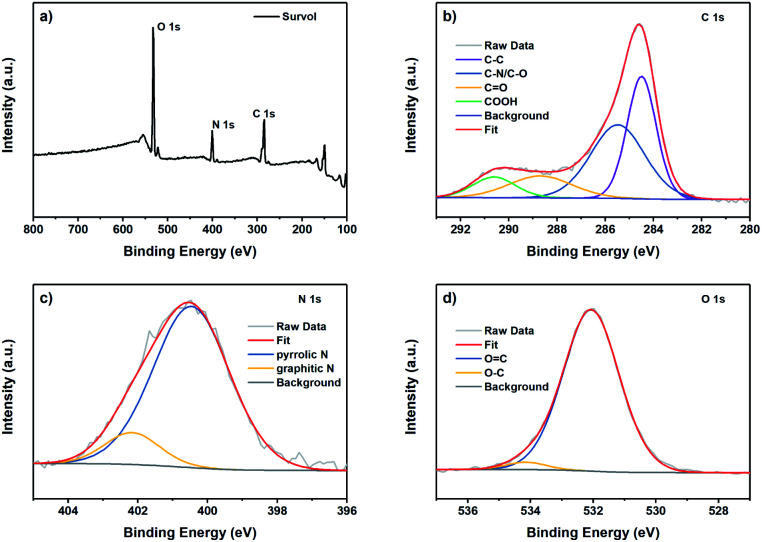
XPS spectra of the CDs synthesized from brewery waste: full spectrum (a) and the deconvoluted XPS peaks of C 1s (b), N 1s (c) and O 1s (d).

X-ray photoelectron spectroscopy (XPS) was carried out to evaluate the CDs' surface functional state and chemical composition. As shown in Fig. 3a, three main peaks can be identified around 284, 400, and 531 eV, attributed to C 1s, N 1s, and O 1s, respectively. Thus, the elemental composition of the as-synthesized CDs is mainly constituted of carbon, oxygen and nitrogen. The C 1s peak can be deconvoluted into four peaks that can be assigned to the carbon sp^2^ hybridization in the form of C–C or C=C at 284.5 eV, to the carbon sp^3^ hybridization in the form of C–O and C–N at 285.6 eV, to C=O at 288.6 eV, and COOH at 290.5 eV (Fig. 3b).^45^ The N 1s spectrum shows two main peaks centered at 400.5, and 402.5 eV, attributed to pyrrolic and graphitic nitrogen, respectively (Fig. 3c). The pyrrolic-N, which represented the main component, is known to enhance the photoluminescent intensity of N-doped CDs.^46^

The O 1s spectrum can be deconvoluted into two peaks at 532.0 and 534.1 eV, attributed to C=O and C–O groups, respectively (Fig. 3d). It has been reported that the emission of the CDs is seriously affected by the degree of surface oxidation rather than the particle size.^47^ XPS results complement the FTIR results, thereby confirming that the surface of CDs contains a high content of oxides and that the CDs are successfully doped with the N element.

### 3.2 Optical properties of CQDs

**Fig. 4:**
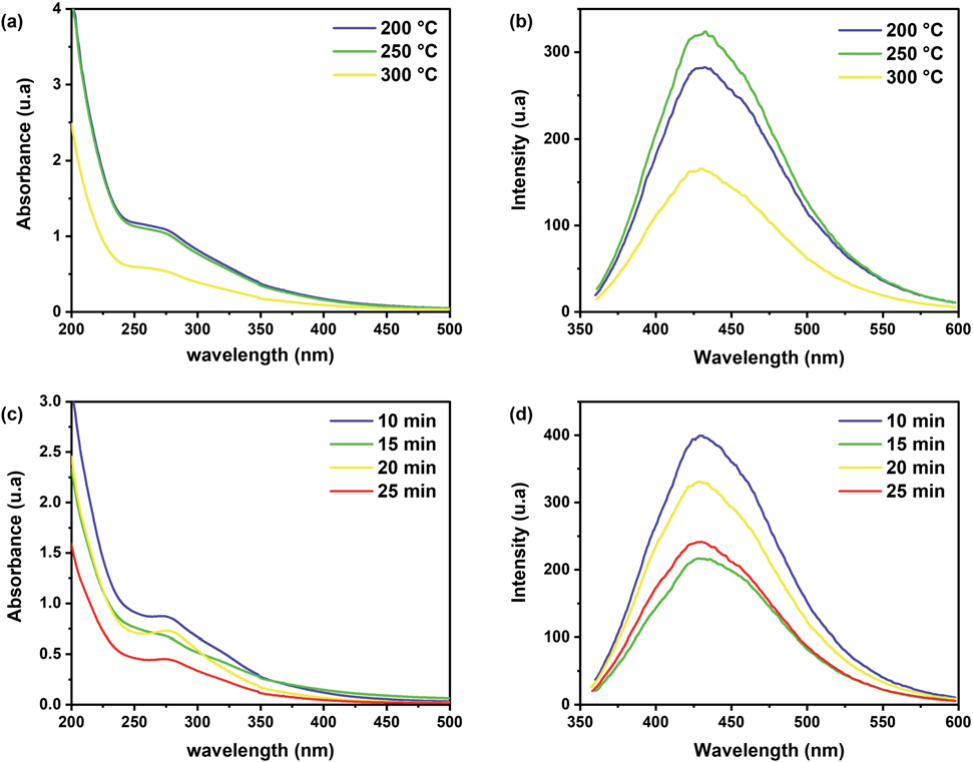
(a) UV-Vis absorption and (b) PL emission spectrum of CD samples at different temperatures (*λ*_ex_ = 350 nm), (c) UV-Vis absorption and (d) PL emission spectrum of CDs samples at different reaction times (*λ*_ex_ = 350 nm). The concentration for each sample was fixed at 1 mg ml^−1^.

To identify the best experimental conditions to obtain CDs and understand the effect of process parameters, such as time and temperature, on the optical properties of brewery waste-based CDs, comparative samples were prepared by varying one parameter at a time. In the first step, three different microwave temperatures were tested, with a fixed reaction duration of 10 minutes. The thermal degradation of brewery wastes, like all lignocellulosic materials, starts from 150 °C with a significant mass loses at temperatures of 200–500 °C.^37^ Consequently, three temperatures were considered: 200, 250, and 300 °C, while above this value, the mass loss increased rapidly. An apparent effect of reaction temperature on the optical properties can be observed in Fig. 4a and b. The UV-Vis absorption spectra of the obtained CDs at the same concentrations (1 mg ml^−1^) present an intense absorption band at 275 nm and an extended tail to the visible region, attributed to π–π* transition of aromatic C=C and n → π* transition of the C=O/C=N, respectively.^48^ Higher absorption in the visible range can be found for the samples prepared at 200 °C and 250 °C while increasing the reaction temperature further caused a signal decrease. For the PL spectra obtained at *λ*_ex_ = 350 nm, only a slight increase in the intensity of the emission was observed. The fluorescence intensity of CDs increased with increasing temperature at 250 °C, but it decreased at 300 °C due to the change in the chemical structure that occurred during the thermal degradation, as reported in other works.^49^ The PL quantum yield (QY) of the CDs prepared at 250 °C for 10 min was calculated to be around 14%. Therefore, as the optimum temperature, 250 °C was selected for the following experiments.

To investigate the effect of the reaction time on the CDs' properties, comparative samples were synthesized using microwave times of 10, 15, 20 and 25 min, at a fixed temperature of 250 °C. Although no changes in the shape of the spectra and no shift in emission peak positions were observed (Fig. 4c and d), the fluorescence intensity was higher at 10 min. Therefore, for the remainder of the experiments, the sample obtained with a reaction time of 10 min at 250 °C is considered.

**Fig. 5:**
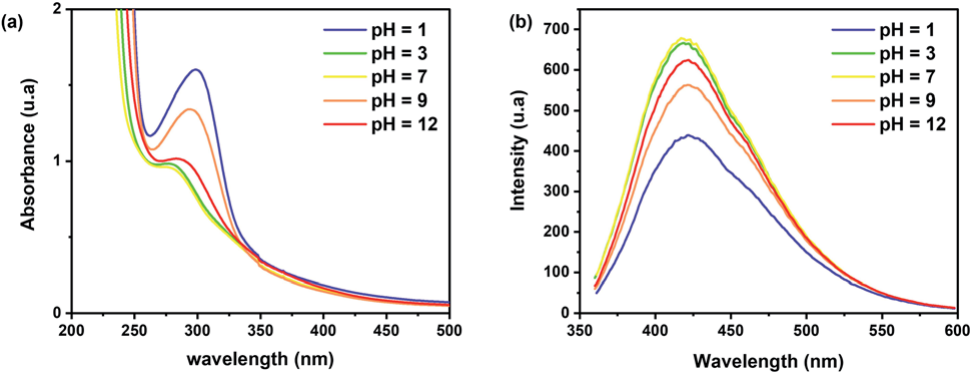
(a) UV-Vis absorption and (b) PL emission spectrum of CDs samples at different pH values (*λ*_ex_ = 350 nm, CDs prepared at 250 °C for 10 min).

Previous studies reported that the pH of the solution-containing CDs affects their PL intensity.^45^ The presence of H^+^/OH^−^ ions changes the functional groups by disrupting or even prohibiting the electronic transition of some surface defects.^50^ To observe the pH sensitivity on the photoluminescent behaviour, different solutions of CDs in distilled water were prepared by varying pH at levels of 1, 3, 7, 9, and 12. The UV-Vis absorption spectrum shows an absorption band located at 280–300 nm (Fig. 5a), which can be assigned to the π → π* transition of the C=C bonds.^51^ By varying the pH values, the surface electrostatic charge of CDs changes with the protonation and deprotonation of the carboxyl groups of the CDs, thereby shifting the absorption peak from 300 to 280 nm.^52^Fig. 5b shows that the maximum emission intensity is observed for a pH = 7, which increases the potential of brewery waste-based CDs to be used in biomedical applications that require neutral pH compatibility to create physiological conditions and a high hydro solubility.^48,53^ However, in both highly acidic and basic mediums, there is a decrease in the fluorescence intensity. This behaviour can be due to the chemical nature of the surface moieties found on the CDs, which impact their electronic properties.^51^

### 3.3 Carbon quantum dots as metal sensors based: fluorescence quenching

**Fig. 6:**
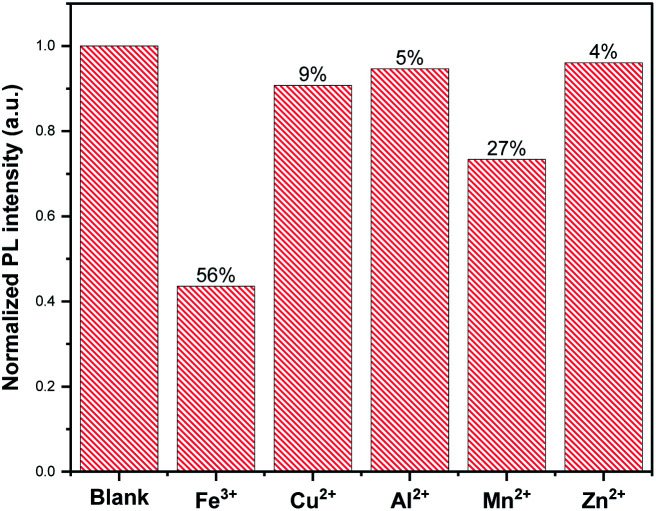
Comparison of the change of CDs fluorescence intensities in the absence and presence of different metal ions at 500 μM.

CDs can be used for the efficient detection of heavy metal in water, such as Hg^2+^,^3^ Pb^2+^ ^31^ and Ag^+^.^54^ In the present work, to evaluate the sensitivity of beer waste-derived CDs prepared different metal ions, *viz.*, Cu^2+^, Fe^3+^, Zn^2+^, Mn^2+^ and Al^2+^, were tested. In the presence of metal ions, the CDs emission was quenched. In particular, a high sensitivity to ferric ions with more than 56% decrease in the CDs' emission intensity (Fig. 6) was observed. However, a slight PL decrease of 5% and 4% was observed by adding Al^2+^ and Zn^2+^, respectively.

**Fig. 7:**
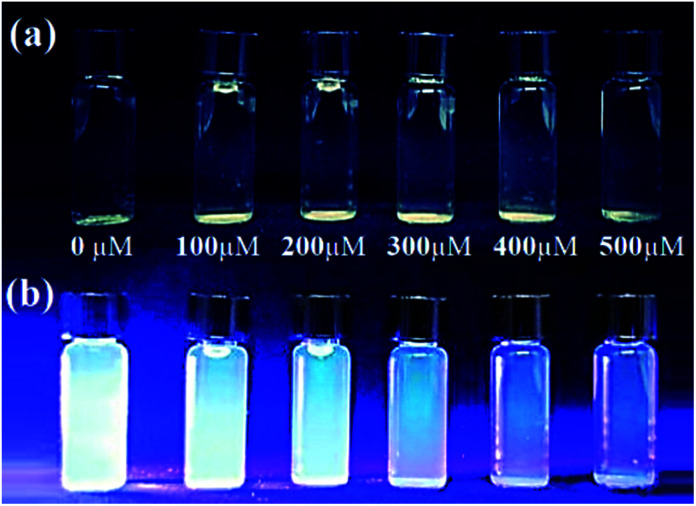
Visual decrease in intensity of the CD blue luminescence with the addition of Cu^2+^ ions under daylight (a) and under UV lamp (b).

**Fig. 8:**
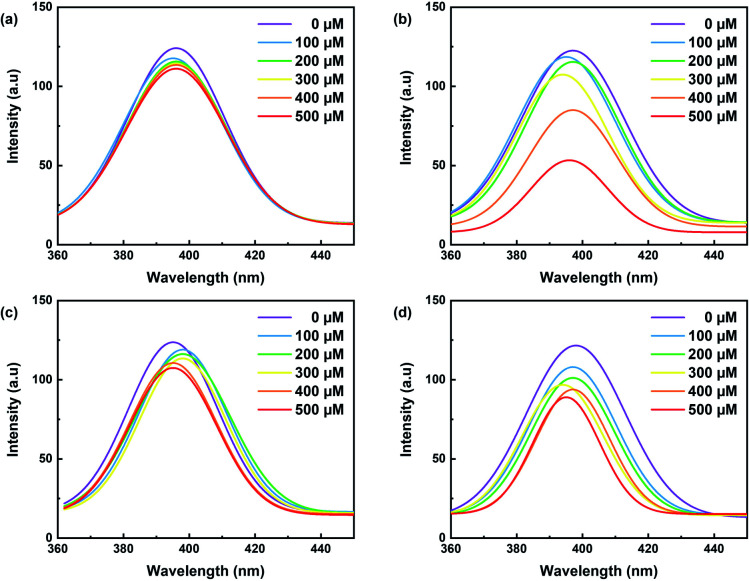
Fluorescence spectra of carbon dots with increasing concentrations of (a) Cu^2+^ (b) Fe^3+^, (c) Zn^2+^ and (d) Al^2+^ (*λ*_ex_ = 350 nm for all samples).

**Fig. 9:**
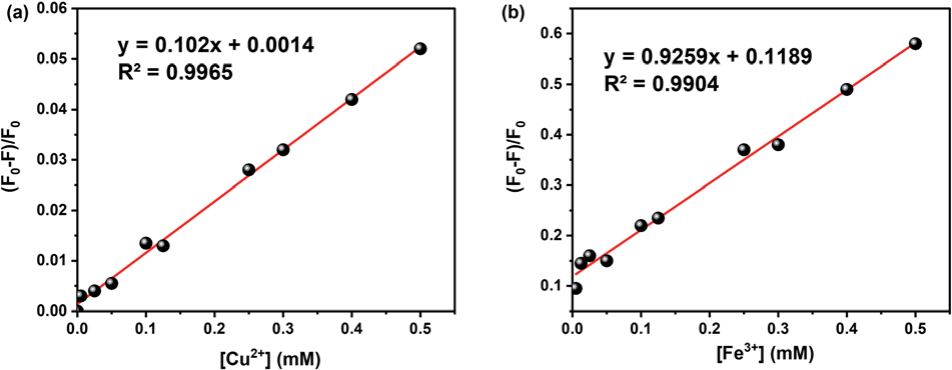
The linear relationship between fluorescent intensity and concentration of: (a) Cu^2+^ and (b) Fe^3+^.

The effect of metal ion content on the PL intensity of CDs was investigated. As expected, the PL decreases with the increasing concentration of metal ions, as illustrated in Fig. 7. Indeed, Fig. 8a showed a gradual decrease in the fluorescence intensity by increasing the Cu^2+^ ion concentration from 0 to 500 μM. When Cu^2+^ ions were added to an aqueous solution of CDs, the PL decreased proportionally with the concentration of copper. The relationship is linear with the correlation function (*F*_0_ − *F*)/*F*_0_ = 0.102 [Cu^2+^] + 0.0014 (with *R*^2^ = 0.9965) (Fig. 9a). This quenching effect resulted from the interaction between copper ions and some surface functional groups (such as hydroxyl, carboxyl and amino groups) of the CDs.^55^

Fe^3+^ ions were also tested in several concentration intervals from 0 to 500 μM. The fluorescence intensity of the CDs solution varies by approximately 2.1 a.u. (Fig. 8b). This is likely due to the participation of Fe^3+^ ions in electron transfer with the hydroxyl groups on the surface of CDs, as shown in the FTIR spectrum. This electron transfer creates additional emission interference while decreasing the fluorescence signal.^35^Fig. 9b shows the linear correlation between (*F*_0_ − *F*)/*F*_0_ and the concentration of Fe^3+^ was observed (*F*_0_ − *F*)/*F*_0_ = 0.9259 [Fe^2+^] + 0.1189 (*R*^2^ = 0.9904) and the limit of detection was 0.05 μM. This value compares favourably with other CDs' detection limits, especially designed for sensing iron ions in water for environmental analysis.^35^ Solutions containing iron ions present a high-quenching effect on the CD's fluorescence. The variation of intensity was minor for the other metal ions, *viz.*, Zn^2+^ and Al^2+^ as illustrated in Fig. 8c and d.

Overall, the CDs produced from microbrewery waste were more efficient for detecting Fe^3+^ ions than Zn^2+^, Al^2+^ and Cu^2+^. The present investigation shows that the brewery waste-derived CDs could be used as metal tracers for select metals.^56^ The study of the interference of the metal ions will be conducted in another study.

## 4 Conclusions and perspectives

The use of microbrewery waste as a precursor for CDs highlights both an eco-responsible approach to waste management and alternative raw material for synthesizing CDs in a perspective of a circular economy. Importantly, this lignocellulosic biomass waste presented a promising precursor for producing N-doped CDs rich in surface functional groups like hydroxyl and carboxyl. The prepared CDs exhibited excellent optical properties (quantum yield of 14%) and a pH-dependent luminescence behavior with a maximum emission at neutral pH. Finally, these novel CDs were tested for metal detection. They were found to be sensitive to the variation in Cu^2+^, Fe^3+^, Zn^2+^ and Al^2+^ ion concentration. Research on CDs is in dynamic progress, and the present results are promising. This warrants further investigation to enhance the performance and the quality of the doped CDs from bio-sources. The next steps are to characterize the CD from brewery waste beyond a proof-of-concept. Such CDs have the potential to become sophisticated sensors for detecting other anions or molecules in different aqueous solutions and living cells.

## Conflicts of interest

There are no conflicts to declare.

## Supplementary Material
